# Case report: Pylorus-preserving pancreatoduodenectomy for focal congenital hyperinsulinism in a 5-month-old baby

**DOI:** 10.3389/fsurg.2022.1085238

**Published:** 2023-01-30

**Authors:** Gionata Spagnoletti, Zoe Larghi Laureiro, Alberto Maria Fratti, Arianna Maiorana, Maria Carmen Garganese, Milena Pizzoferro, Carlo Dionisi-Vici, Marco Spada

**Affiliations:** ^1^ Division of Hepatobiliopancreatic Surgery, Liver and Kidney Transplantation, Ospedale Pediatrico Bambino Gesù IRCCS, Roma, Italy; ^2^ Division of Metabolic Diseases, Department of Pediatric Specialties, Ospedale Pediatrico Bambino Gesù IRCCS, Roma, Italy; ^3^ Nuclear Medicine, Imaging Department Ospedale Pediatrico Bambino Gesù, IRCCS, Roma, Italy

**Keywords:** congenital hyperinsulinemic hypolglycemia (CHI), pylorus-preserving pancreaticoduodenectomy (PPPD), pediatric surgery, hypoglycaemia, insulin dysregulation

## Abstract

**Background:**

In focal congenital hyperinsulinism (CHI), surgery is the gold standard of treatment, even for lesions localized in the head of the pancreas. We report the video of the pylorus-preserving pancreatoduodenectomy performed in a five-month-old child with focal CHI.

**Operative technique:**

Baby was placed in the supine position with both arms outstretched to the up. After transverse supraumbilical incision and mobilization of ascending and transverse colon, exploration and multiple biopsies of the tail and the body of the pancreas ruled out multifocality. Pylorus-preserving pancreatoduodenectomy was performed according to the following steps: extended Kocher maneuver, followed by retrograde cholecystectomy and common bile duct isolation; division of the gastroduodenal artery and of the gastrocolic ligament; division of the duodenum, Treitz ligament and jejunum; transection of the pancreatic body. The reconstructive time was with: pancreato-jejunostomy; hepaticojejunostomy; pilorus-preserving antecolic duodeno-jejunostomy. The anastomoses were accomplished with synthetic absorbable monofilament sutures; two drains were placed close to the biliary and pancreatic anastomoses and to the intestinal anastomosis, respectively. Total operative time was 6 h, with no blood loss and/or intra-operative complications, immediate normalization of blood glucose levels and discharge from surgical ward 19 days after surgery.

**Conclusions:**

Surgical treatment of medical unresponsive focal forms of CHI is feasible in very small children: it is mandatory to refer the baby to a high-volume centre for a multidisciplinary management involving hepato-bilio-pancreatic surgeons and experts in metabolic disease.

## Introduction

Congenital Hyperinsulinism (CHI) and insulinomas are the two most common causes of medically-refractory hyperinsulinism in infancy (1). In focal CHI, surgery is the gold standard of treatment, even for lesions localized in the head of the pancreas needing a demand surgery (2). We present the video of the pylorus-preserving pancreatoduodenectomy performed in a five-month-old child with focal CHI.

The case reported was performed at Bambino Gesù Children's Hospital, a large pediatric research hospital with more than 600 settled beds, accredited since 2007 by the International Joint Commission, with a division of hepatobiliopancreatic surgery, with high-volume activity including liver transplantation, and a division of metabolic disease.

Informed consent for the collection of images, videos and medical notes was obtained from patient's parents.

A five-month-old baby girl suffered at birth from severe hypoglycaemic crisis, associated with hypertonus and desaturation requiring intubation, umbilical vein catheterization with continuous parenteral infusion of glucose, up to 13.5 mg/kg/min and increasing doses of diazoxide therapy at maximum dosage. Nevertheless, she presented recurrent hypoglycaemia, therefore octreotide was started up to 20 mcg/kg/day, subcutaneously.

Molecular analysis revealed a mutation of the beta-islet cell potassium-ATPase (KATP) channel, inherited from the father, supporting the diagnosis of CHI. At the age of three months, the 18F-Fluoro-L-DOPA PET-CT scan demonstrated a focal (diameter 3 cm) hyper-uptake in the head of the pancreas. Due to persistence of recurrent hypoglycemia and vomiting, at the age of 5 months and with a body weight of 7.4 kg she underwent to surgical treatment with partial pancreatectomy.

The video reports the pylorus-preserving pancreatoduodenectomy performed in the small baby.

Postoperative period was uneventful, the patient was transferred to the surgical ward after 24 h of monitoring in Intensive Care Unit. Doppler ultrasound excluded abdominal collections and abdominal drainages were removed in post-operative days VII and X, respectively. Starting from the immediate post-operative period, she maintained normal blood glucose levels with standard glucose infusion (5% glucose). Enteral feeding was progressively reintroduced. Final histological report confirmed a focal lesion of ß-cell adenomatosis of the pancreatic head, surrounded by normal pancreatic tissue.

### Operative technique

Baby was placed in the supine position with both arms outstretched to the up to allow for a Thompson retractor (Surgi-One Medical Technologies, Burlington, Canada) placement. After a transverse supraumbilical incision, the ascending and transverse colon were mobilized, with division of the gastrocolic ligament. CHI multifocality was ruled out with exploration of the whole pancreas and multiple biopsies of the tail and the body: the intraoperative histological examination showed normal pancreatic parenchyma. The video will be in the Supplementary Materials.

We first tried a duodenum preserving resection of the head of the pancreas, enucleating the area of the head and uncinate process of the pancreas where the PET-CT showed the presence of the focal CHI. The intraoperative histological examination confirmed the presence of ß-cell adenomatosis with positive resection margins. Since a further extension of the wedge resection would compromise the integrity of the Wirsung, we opted for a pancreatoduodenectomy to guarantee a complete removal of the disease.

The entire duodenum was detached from the retroperitoneum and inferior vena cava by Kocher maneuver and the Treitz ligament was dissected. The first portion of the duodenum and the first jejunal loop were stapler transected, after ligation and division of the gastroepiloic pedicle. The gallbladder was separated from the liver, the extrahepatic bile duct was mobilized and divided above the confluence of the cystic duct. The gastroduodenal artery was isolated and divided close to its origin from the common hepatic artery; the pancreatic isthmus was separated from the anterior aspect of the portal vein and the pancreas divided at this level by means of bipolar and cold blade, not to jeopardize Wirsung's vascularization. The head of the pancreas was thereafter detached from the right lateral aspect of the portal vein with ligation and division of some small branches. The retroportal plate was divided, with identification and preservation of the superior mesenteric artery, completing the pancreatico-duodenectomy. Intraoperative pathology confirmed the absence of ß-cell adenomatosis on the resection margin.

**Figure 1:**
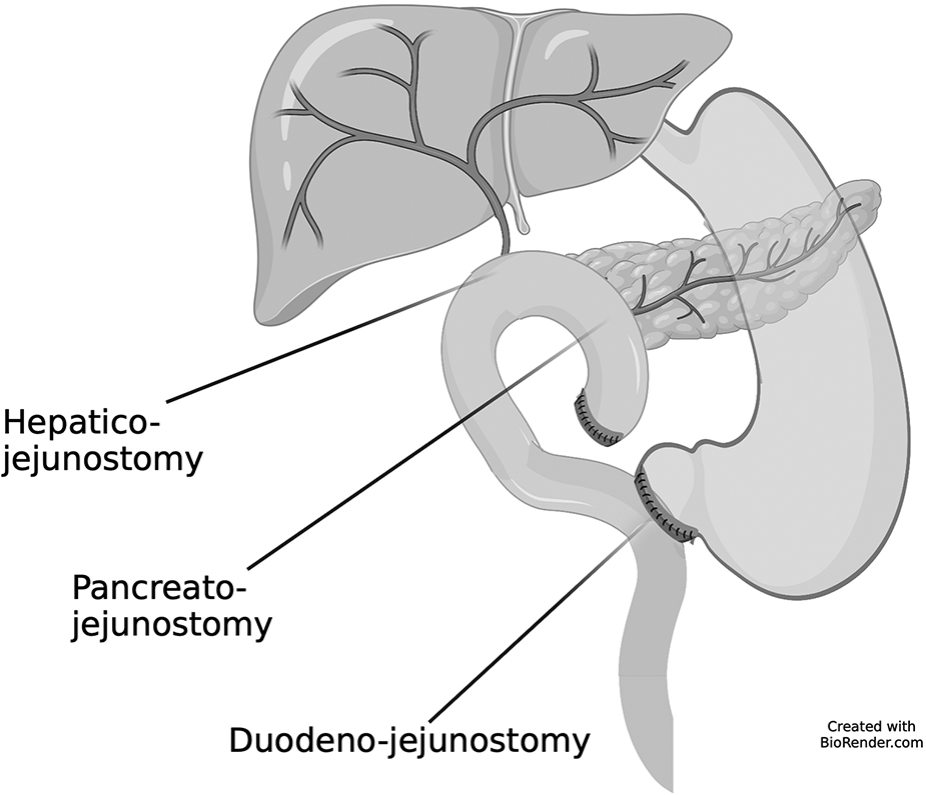
Schematic representation of the post-operative anatomical condition.

The reconstructive time started with an end-to-side pancreato-jejunostomy. The first jejunal loop was rotated behind the mesenteric root and pullet into the sovramesocolic area; because of the small caliber of the Wirsung duct, which was anyway localized with a small probe in order to avoid suturing it, a double layer anastomosis with interrupted absorbable 5–0 and 4–0 stitches between the pancreatic cut surface and a small hole in the jejunal loop was accomplished. Hepaticojejunostomy was created 10 cm distally to the pancreatic anastomosis with an end-to-side single layer anastomosis with synthetic absorbable 6–0 monofilament suture. After pyloroplasty with Hegar dilators, antecolic end-to-side duodenojejunostomy was created, 50 cm distally to the hepaticojejunostomy, with a double layer anastomosis with synthetic absorbable 5–0 monofilament suture. Figure 1. Two drains were placed close to the biliary and pancreatic anastomoses and to the intestinal anastomosis, respectively.

Total operative time was 6 h, with no blood loss and/or intra-operative complications.

Among the many reconstruction methods for pancreatoduodenectomy, we chose to perform a pylorus-preserving pancreatoduodenectomy due to the intra-operative aspect of the pancreas that was not excessively soft and/or friable. In addition, using a 3.5 × loupe magnification, the Wirsung duct was seen clearly and specified with confidence. In these conditions, in our opinion, it is safe to perform Wirsung-jejunum and second layer pancreas-jejunum. This, in our experience, represents the best choice to reduce the risk of fistula.

### Discussion

Stanley and Baker in 1976 proposed the first definition of CHI outlining the diagnostic criteria: hypoglycemia, hypoketonemia, hypo-fatty acidemia, hyperglycemic response to glucagon (3).

The incidence of CHI is estimated at 1 in 40,000 live births in the general population and it reaches 1 in 2,500 in populations with high rates of consanguinity (4, 5).

CHI is caused by genetic mutations and at least eleven monogenic forms of CHI have been identified, but in up to 50% of patients, the cause of CHI remains unknown, suggesting the role of additional genetic loci (6). The most common and severe form of monogenic CHI comes from mutations affecting the potassium-ATPase (KATP) channel of the ß-cell plasma membrane. Diffuse-type CHI is caused by two mutant KATP alleles, resulting in disease throughout the entire pancreas. Focal-type CHI is due to a paternally-inherited KATP mutation and the loss of heterozygosity in a focused area of the pancreas during foetal development, resulting in a localized region of hyperactive insulin release (7).

In our patient we found a single variant c.3989–2A > G in ABCC8. The variant, in heterozygosity and paternal segregation, was not present in the general population frequency database (gnomAD (8)), it was not described in the literature and, according to the ACMG guidelines (9), was described as a pathogenitc variant.

The 18F-Fluoro-L-DOPA PET-CT scan is considered the diagnostic gold standard for the detection of focal lesions, yielding a sensitivity from 75% to 100% and specificity from 88% to 100% (10). In the described case we confirmed the single hypercaptation at the head of the pancreas. Furthermore, CT images are useful for planning the surgical strategy: to assess the relationship of the lesion with the portal vein and the superior mesenteric artery; to exclude anatomical variants, especially the presence of a right hepatic artery from the superior mesenteric artery.

Inactivating KATP mutations are usually unresponsive to diazoxide. In these patients, medical therapeutic target is to maintain plasma glucose >70 mg/dl (11), to avoid neurological outcomes. To reach this threshold, high continuous intravenous infusions of glucose, plus eventual i.v. glucagon, frequent or continuous enteral feeding, and administration of octreotide are combined (12).

In 2009 Adzick et al. (13, 14), reported extensive experience on 500 neonates and children who underwent pancreatectomy, between 1998 and 2018, to treat congenital hyperinsulinism in its various forms.

The authors suggested near-total pancreatectomy for diffuse-type CHI and local resection for focal types, aimed at removing as much of the diseased pancreas as possible in a safe manner and they concluded that a multidisciplinary team is required to manage these patients involving hepatobiliopancreatic surgeons, metabolic disease experts, genetics, radiology and pathologists.

According to Adzick, we ruled out a diffuse pancreatic disease with intraoperative biopsies and then we tried to perform a wedge resection. Since intraoperative resection margins were positive, we opted for a pancreatoduodenectomy to guarantee a complete removal of the disease.

## Conclusions

We presented the intraoperative video of a pancreatoduodenectomy performed in a 5-month-old baby affected by focal CHI of the head of the pancreas non-responsive to medical treatment, detailing the techniques adopted in the different phases of the procedure.

Operation was uneventful and the baby achieved and maintain euglycemia soon after surgery.

In our experience, as suggested by other authors, successful surgical treatment of medical refractory CHI is feasible in very young children: it is mandatory to refer the baby to a high-volume centre for a multidisciplinary involving hepatobiliopancreatic surgeons and experts in metabolic disease.

## Data Availability

The original contributions presented in the study are included in the article/Supplementary Material, further inquiries can be directed to the corresponding author/s.
